# Subtraction-free and bisulfite-free specific sequencing of 5-methylcytosine and its oxidized derivatives at base resolution

**DOI:** 10.1038/s41467-021-20920-2

**Published:** 2021-01-27

**Authors:** Yibin Liu, Zhiyuan Hu, Jingfei Cheng, Paulina Siejka-Zielińska, Jinfeng Chen, Masato Inoue, Ahmed Ashour Ahmed, Chun-Xiao Song

**Affiliations:** 1 Ludwig Institute for Cancer Research, Nuffield Department of Medicine, University of Oxford, Oxford, OX3 7FZ UK; 2 Target Discovery Institute, Nuffield Department of Medicine, University of Oxford, Oxford, OX3 7FZ UK; 3 Ovarian Cancer Cell Laboratory, MRC Weatherall Institute of Molecular Medicine, University of Oxford, Oxford, OX3 9DS UK; 4 Nuffield Department of Women’s & Reproductive Health, University of Oxford, Oxford, OX3 9DU UK; 5 Present Address: Exact Sciences Innovation, Innovation Building, Oxford, OX3 7FZ UK

**Keywords:** DNA sequencing, DNA methylation, DNA methylation

## Abstract

Although various methods have been developed for sequencing cytosine modifications, it is still challenging for specific and quantitative sequencing of individual modification at base-resolution. For example, to obtain both true 5-methylcytosine (5mC) and true 5-hydroxymethylcytosine (5hmC) information, the two major epigenetic modifications, it usually requires subtraction of two methods, which increases noise and requires high sequencing depth. Recently, we developed TET-assisted pyridine borane sequencing (TAPS) for bisulfite-free direct sequencing of 5mC and 5hmC. Here we demonstrate that two sister methods, TAPSβ and chemical-assisted pyridine borane sequencing (CAPS), can be effectively used for subtraction-free and specific whole-genome sequencing of 5mC and 5hmC, respectively. We also demonstrate pyridine borane sequencing (PS) for whole-genome profiling of 5-formylcytosine and 5-carboxylcytosine, the further oxidized derivatives of 5mC and 5hmC. This work completes the set of versatile borane reduction chemistry-based methods as a comprehensive toolkit for direct and quantitative sequencing of all four cytosine epigenetic modifications.

## Abstract (web summary)

Specific and quantitative sequencing of cytosine modifications is challenging at base-resolution. Here the authors present TAPSβ and CAPS for subtraction-free whole genome sequencing of 5mC and 5hmC.

## Introduction

The primary DNA sequence of the four-letter alphabets G, C, A, and T forms the genetic information of life on earth. Chemical modifications of DNA bases do not change the underlying sequence, but instead carry an extra layer of information. The first discovered 5-methylcytosine (5mC) is the most studied modified base, and it plays crucial roles in a broad range of biological processes from gene regulation to normal development^1^ and is regarded as the fifth base. 5-Hydroxymethylcytosine (5hmC) is converted from 5mC by the ten-eleven translocation (TET) family of dioxygenases^2^; it is enriched in neuronal cells^3^ and regarded as the sixth base. Further successive TET oxidation results in 5-formylcytosine (5fC) and 5-carboxylcytosine (5caC)^4,5^, which exist at much lower abundances in the mammalian genome and are regarded as intermediates in the thymine DNA glycosylase (TDG)-base excision repair (BER) active demethylation pathway^5^. Emerging evidence indicates the stability of these DNA demethylation intermediates^6^ as well as potential functional roles^7^.

Detection and analysis of cytosine modifications has been an intriguing challenge for chemists as well as other scientists. Traditionally, bisulfite sequencing (BS) has been the gold standard for base-resolution and quantitative analysis of 5mC and 5hmC^8^. Modified BS has also been developed for specific sequencing of 5mC (oxidative bisulfite sequencing, oxBS-seq)^9^ or 5hmC (TET-assisted bisulfite sequencing, TAB-seq)^10^. These methods, however, all involve harsh bisulfite treatment, which degrades up to 99% of the DNA^11^, and reduces sequence complexity by converting unmodified cytosine (~95% of the total cytosine in the human genome) to thymine (T). Recently, bisulfite-free quantitative base-resolution methods have emerged and showed significant advantages over BS^12^. Among them, APOBEC-coupled epigenetic sequencing (ACE-seq, which detects 5hmC)^13^ and Enzymatic Methyl-seq (EM-seq, which detects 5mC + 5hmC)^14^ use an enzymatic deamination step to replace the bisulfite deamination step. While these methods solve the DNA damage issue, they still suffer from the indirect detection issue of BS by converting unmodified cytosine to T. Recently, we developed TET-assisted pyridine borane sequencing (TAPS) based on a pyridine borane reductive decarboxylation and deamination chemistry^15,16^. In TAPS, 5mC and 5hmC are oxidized by TET proteins to 5caC and reduced to dihydrouracil (DHU) by pyridine borane. DHU is then amplified and sequenced as T during sequencing. TAPS is nondestructive and detects 5mC + 5hmC directly, and it shows improved sequence quality, mapping rate, and coverage compared to BS^15^.

5mC and 5hmC provide distinct and antagonistic epigenetic information: 5mC usually marks repressed genes and 5hmC generally marks expressed genes^17^. To elucidate the interplay between 5mC and 5hmC in various biological processes, it is necessary to distinguish the two modifications. To do that, two assays (e.g. BS and oxBS-seq, BS and TAB-seq, or EM-Seq and ACE-seq) need to be performed and a subtraction between the two assays is usually required to obtain both the true 5mC and true 5hmC information (e.g. BS minus oxBS-seq to get 5hmC, BS minus TAB-seq to get 5mC, or EM-Seq minus ACE-seq to get 5mC)^9,10,13^. However, subtraction may introduce negative values because of random sampling or systematic error in each experiment and suffer from accumulation of noise from multiple assays, which increases the need for higher sequencing depth^18^ as well as more effort to perform read filtering and apply statistical tests^19^. A subtraction-free approach where two assays (e.g. oxBS-seq and TAB-seq) can read out the true 5mC and true 5hmC information directly is desirable. However, so far, no bisulfite-free methods have been shown to deliver that. Previously, we demonstrated the proof-of-principle that the versatility of the borane reduction chemistry for direct and quantitative sequencing of individual cytosine modification on model DNA with Sanger sequencing^15^. In this study, we further optimize and demonstrate these methods for whole-genome applications, including TAPS with β-glucosyltransferase (βGT) blocking (TAPSβ) and chemical-assisted pyridine borane sequencing (CAPS) for whole-genome subtraction-free 5mC-specific and 5hmC-specific sequencing, respectively; and pyridine borane sequencing (PS) for whole-genome sequencing of 5fC and 5caC.

## Results

### TAPSβ for bisulfite-free 5mC-specific sequencing

**Fig. 1:**
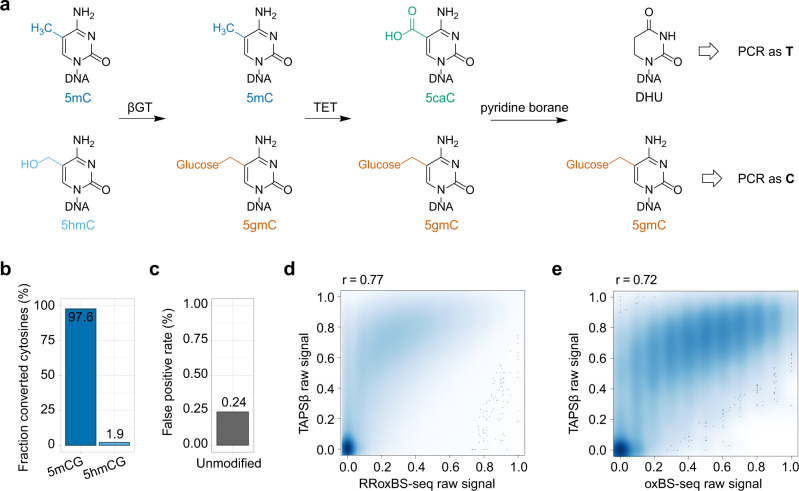
TAPSβ for bisulfite-free 5mC-specific sequencing. **a** Schematic demonstration of TAPSβ. **b** Conversion rates of TAPSβ at known 5mCG or 5hmCG positions from CpG-methylated lambda DNA or synthetic spike-in. **c** False-positive rate of TAPSβ from 2-kb-unmodified spike-in. **d** Correlation analysis between TAPSβ and published RRoxBS-seq dataset at CpGs with the minimal depth of 10. The color scale represents density. **e** Correlation analysis between TAPSβ and published oxBS-seq dataset at CpGs with the minimal depth of 10. The Pearson’s *r* is shown at the top. The raw signal for each CpG was calculated as the ratio between C and the sum of C and T.

To realize 5mC-specific sequencing, we used βGT, which is widely used for selective labeling of 5hmC with glucose that enables 5hmC pull-down^20^ and protection from TET oxidation^10^ or APOBEC deamination^13^. We utilized this simple and robust reaction to block 5hmC and then performed TET oxidation and borane reduction on 5mC (Fig. 1a)^15^. We applied TAPSβ on mouse embryonic stem cells (mESCs) genomic DNA (gDNA) and validated with spike-in controls with known modifications by high-throughput sequencing. High 5mC conversion rate (97.6% in CpG-methylated lambda DNA, Fig. 1b) and low false-positive rate (0.24% conversion rate on unmodified C, Fig. 1c) were achieved in TAPSβ, which are close to previous TAPS results (96.5% and 0.23%, respectively)^15^. 5hmC showed only 1.9% conversion rate in TAPSβ (Fig. 1b) compared to 89.1% in TAPS^15^. The other two minor cytosine modifications 5fC and 5caC also showed high conversion rate (84.9% and 94.4% respectively, Supplementary Table 1); however, they were ignored in following data analysis due to the negligible amounts existed in the mammalian genome (<0.002% of total cytosine)^4^. Similar to TAPS^15^, TAPSβ showed excellent sequencing quality scores at cytosine/guanine (Supplementary Fig. 1). We observed good correlation between TAPSβ and published 5mC data of mESCs by reduced representation oxBS-seq (RRoxBS-seq)^21^ (Pearson’s *r* = 0.77, Fig. 1d) and whole-genome oxBS-seq^22^ (Pearson’s *r* = 0.72, Fig. 1e). In comparison, Pearson correlation coefficients between the reported four RRoxBS-seq replicates were 0.79–0.80 (ref. ^21^). The discrepancy between TAPSβ and oxBS-seq is likely caused by biological differences, e.g. cell line passages^9^, rather than systematic bias, given the high correlation between TAPS and TAPSβ using the same biological sample (Supplementary Fig. 2). TAPSβ showed much improved sequencing quality evidenced by higher mapping rate (90.7%, Supplementary Table 2) than RRoxBS-seq^21^ (66.2–68.2%) and oxBS-seq^22^ (21.4–26.1%). Notably, TAPSβ is the first and only bisulfite-free, base-resolution, and quantitative 5mC-specific sequencing method.

### CAPS for bisulfite-free 5hmC-specific sequencing

**Fig. 2:**
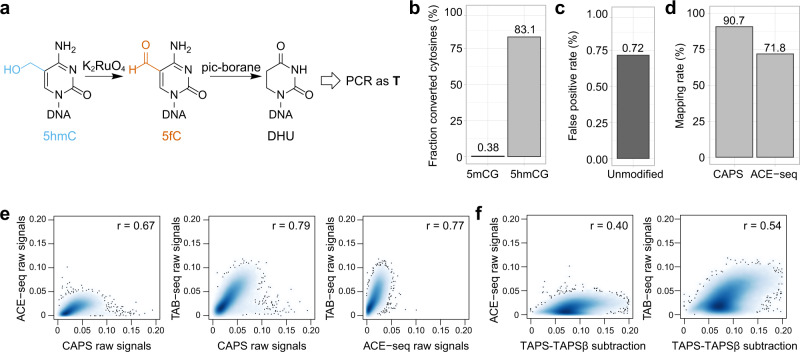
CAPS for bisulfite-free 5hmC-specific sequencing. **a** Schematic demonstration of CAPS. **b** Conversion rates of CAPS at known 5mCG or 5hmCG positions from CpG-methylated lambda DNA or synthetic spike-in. **c** False-positive rate of CAPS from 2-kb-unmodified spike-in. **d** Fraction of all sequenced read pairs in CAPS and ACE-seq mapped to the reference mouse genome. **e** Correlation density plot between CAPS, TAB-seq, and ACE-seq in 10-kb bins. The color scale represents density. **f** Correlation density plot between TAPS−TAPSβ subtraction and TAB-seq or ACE-seq in 10-kb bins.

To enable 5hmC-specific sequencing, we turned to chemical oxidization of 5hmC to 5fC, which can also be converted to DHU by borane reduction (Fig. 2a). In our proof-of-principle study, we used potassium perruthenate (KRuO_4_) previously used in oxBS-seq as the oxidant, which is known to cause DNA damage^9^. In this study, we utilized potassium ruthenate (K_2_RuO_4_), which was used in chemical-assisted C-to-T conversion of 5hmC sequencing (hmC-CATCH) and reported to be more oxidative and less DNA damaging than KRuO_4_ (ref. ^23^). We optimized the K_2_RuO_4_ oxidation protocol for CAPS as follows: (1) Commonly used double-strand DNA library preparation method was applied instead of the complicated single-strand protocol. (2) A uracil-containing loop-structured NEBNext Adaptor was used in the DNA ligation. Subsequent treatment with USER enzyme (a mix of UDG and Endo VIII) opened the loop, leaving 3ʹ and 5ʹ phosphate ends that could protect the ligated DNA from oxidative damaging^24^. (3) Double oxidation was performed on the ligated DNA by adding additional oxidant to the original oxidation reaction, improving the conversion rate of 5hmC to 5fC from 82.8% to 97.2% measured by HPLC-MS/MS (Supplementary Fig. 3). A limitation of both KRuO_4_ and K_2_RuO_4_ oxidation is that they only work on single-strand DNA. Pyridine borane used in TAPS, which is optimized based on double-strand DNA, only showed 65.8% 5hmC-to-T conversion rate on single-strand DNA while 1.3% C-to-T false-positive rate was observed (Supplementary Fig. 4). Instead, we found that another compound, 2-methylpyridine borane (pic-borane), achieved 83.1% 5hmC-to-T conversion rate (Fig. 2b) and 0.72% false-positive rate on single-strand DNA (Fig. 2c), and therefore was chosen as the reducing agent for CAPS. These numbers are similar to the 5hmC-to-T conversion rate and false-positive rate reported in hmC-CATCH (~80% (without pull-down) and 0.6–1%, respectively)^23^.

Next, we applied CAPS on mESCs and detected 1,762,287 5hmC-modified sites. We compared CAPS with the other two whole-genome base-resolution 5hmC sequencing methods: TAB-seq^10^ and ACE-seq^13^, using published sequencing data from mESCs. Both TAB-seq and ACE-seq utilize βGT to protect 5hmC with a glucose from bisulfite or enzymatic deamination and read it as C after PCR amplification, while converting both unmodified C and 5mC to T. Bisulfite-based TAB-seq shares the same drawbacks as BS, while ACE-seq partially solves the problem by replacing the harsh chemical reaction with mild APOBEC3A enzymatic deamination. However, ACE-seq still suffers from reduced sequence complexity in the converted genome, which results in low mapping rate (Fig. 2d and Supplementary Table 2), low base quality (Supplementary Fig. 5), and uneven coverage (Supplementary Fig. 6). The low base quality in ACE-seq is caused by the unbalanced CG content in the sequencing libraries similar to WGBS^15^, while CAPS avoids depletion of cytosines leading to optimal data quality similar to TAPS and TAPSβ. CAPS outperformed TAB-seq and ACE-seq in these sequencing metrics (Fig. 2d and Supplementary Table 2), while showing good correlation with published dataset (Pearson’s *r* = 0.79 with TAB-seq and 0.67 with ACE-seq, Fig. 2e). On the other hand, 5hmC obtained from TAPS−TAPSβ subtraction showed an abnormal distribution of modification levels with lower correlation (Pearson’s *r* = 0.54 with TAB-seq and 0.40 with ACE-seq, Fig. 2f), demonstrating that the subtraction-free method is superior for 5hmC profiling, especially given that 5hmC exists in much lower abundance than 5mC in most non-neuronal tissues and cell lines^4^, including mESCs (Supplementary Fig. 3a).

**Fig. 3:**
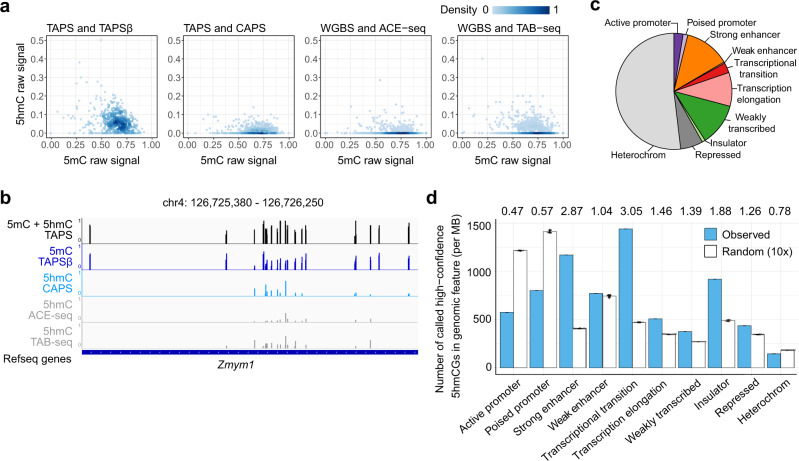
Comparison of CAPS with other methods. **a** Comparison of 5mC and 5hmC levels tiled by 1-kb bins for TAPSβ, CAPS, ACE-seq, and TAB-seq. The levels of unmodified and modified cytosines were estimated by MLML using the direct readout from the method combination shown at the title of each subfigure. **b** Example of genome browser view on chromosome 4 showing CAPS-detected consistent 5hmC sites when compared with ACE-seq and TAB-seq. **c** Pie chart shows the overlap of called 5hmCGs with putative genomic regulatory elements. **d** The relative enrichment of 5hmCG (blue) and random sites (white) at genomic regulatory elements. ‘Random’ consists of ten random samplings. The mean is shown as the bar height and the error bars denote standard deviation (*n* = 10 random sampling events). Each dot denotes one random sampling event. The ratios between observed and random are shown at the top.

To globally benchmark different methods by accounting for 5mC and 5hmC states in mESCs, we established the abundance of both modifications (Fig. 3a). Combination of TAPSβ and CAPS showed a similar pattern to whole-genome BS (WGBS) with TAB-seq or ACE-seq while TAPS−TAPSβ subtraction overestimated 5hmC sites. Examples were plotted to show results from different approaches, demonstrating that CAPS detected consistent 5hmC sites with TAB-seq and ACE-seq (Fig. 3b and Supplementary Fig. 7). The distribution of 5hmC varied across genomic regulatory elements (Fig. 3c)^25–27^, with particular enrichment at enhancers and insulators^28^, where CTCF-binding sites were enriched (Fig. 3d). This result is consistent with previous findings that 5hmCs are enriched in enhancers and CTCF-binding sites^10,23^.

### PS for bisulfite-free 5fC/5caC-specific sequencing

**Fig. 4:**
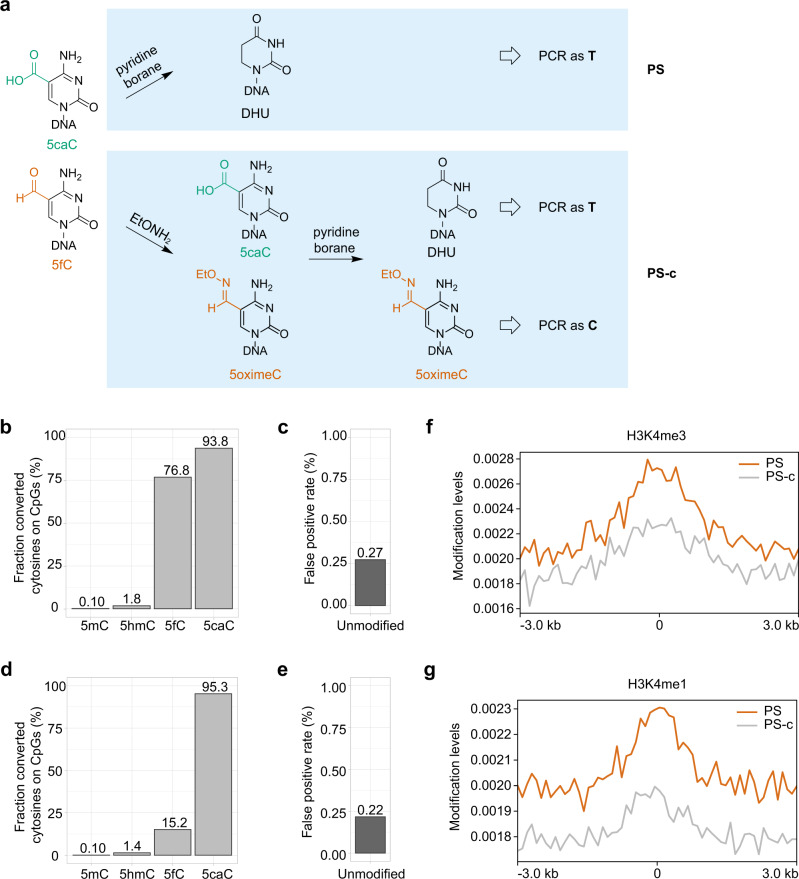
PS for bisulfite-free 5fC/5caC-specific sequencing. **a** Schematic demonstration of PS and PS-c. **b** Conversion rate of PS at known 5mC, 5hmC, 5fC, and 5caC positions in spike-in controls. **c** False-positive rate of PS from 2-kb-unmodified spike-in. **d** Conversion rate of PS-c at known 5mC, 5hmC, 5fC, and 5caC positions in spike-in controls. **e** False-positive rate of PS-c from 2-kb-unmodified spike-in. **f**, **g** Average 5fC/5caC signal in the 6-kb regions flanking the centers of H3K4me3 (**f**) and H3K4me1 (**g**) peaks produced by the ENCODE Project Consortium. On the x-axis, 0 denotes the peak center.

To study the active demethylation pathway, various BS-based^21,29–31^ and bisulfite-free^32^ methods have also been developed to profile 5fC and/or 5caC modifications. The borane reduction chemistry can be used for direct sequencing of 5fC and 5caC, where 5fC and 5caC are converted to DHU by pyridine borane and read as T after PCR amplification (PS, Fig. 4a). We applied this simple approach to the same mESCs gDNA and demonstrated high conversion rate in 5caC spike-in (93.8%, Fig. 4b) and good conversion rate in 5fC spike-in (76.8%). The low false-positive rate (0.27%, Fig. 4c) in PS lowered the requirement for sequencing depth to distinguish the low abundant 5fC/5caC signals from the background^33^. We also developed a method for 5caC-specific sequencing in which 5fC was blocked by *O*-ethylhydroxylamine^29^ before borane reduction reaction, which we termed pyridine borane sequencing for 5caC (PS-c, Fig. 4a). PS-c achieved a low conversion rate of 15.2% on 5fC (Fig. 4d) while the high conversion rate on 5caC (95.3%) and the low false-positive rate (0.22%, Fig. 4e) were not affected. Even with the low false-positive rates of PS and PS-c, it remains challenging to detect 5fC and 5caC in whole-genome sequencing due to their low level. Instead, we focused on regulatory regions^27,34^ and found that 5fC/5caC signals were enriched at H3K4me1, H3K4me3 regions (Fig. 4f, g), promoters and enhancers compared to repressed regions or heterochromatin (Supplementary Fig. 8), which is consistent with previous enrichment-based 5fC sequencing result^29^. Our base-resolution data also revealed the 5fC/5caC modification on *Nanog*, a pluripotency regulator (Supplementary Fig. 9), which was previously reported based on an enrichment-based method^32^. These results suggest that PS can capture the genuine 5fC/5caC signals even in a low 5fC level sample.

Bisulfite and various bisulfite-free methods are known to have systematic differences in sequencing due to different chemistries and detection mode (indirect vs direct)^14,15^. When comparing various modifications, it is therefore ideal to have them derived from the same family of methods to minimize bias. In this study, we present a suite of borane reduction chemistry-based methods for direct and quantitative sequencing of all four cytosine modifications in mESCs, providing a valuable resource for studying DNA modifications in the popular epigenetics model (Table 1). By replacing harsh bisulfite treatment with mild borane reduction reaction, we achieved higher sequencing quality and more comprehensive methylome analysis. Independent identification of 5mC and 5hmC by subtraction-free TAPSβ and CAPS methods could provide insight into the distribution and function of the two modifications. The simple and mild borane reduction sequencing methods PS and PS-c could facilitate studies of the dynamics of active DNA demethylation processes. Together, they offer the most comprehensive solution for epigenetic sequencing of cytosine modifications.

**Table 1:** Base changes in borane reduction chemistry-based methods.

Base	TAPS	TAPSβ	CAPS	PS	PS-c
C	C	C	C	C	C
5mC	**T**	**T**	C	C	C
5hmC	**T**	C	**T**	C	C
5fC	T	T	T	**T**	C
5caC	T	T	T	**T**	**T**

C-to-T transitions marked in bold were recognized as modified sites.

## Methods

### Preparation of spike-in DNA

CpG-methylated lambda DNA was produced from unmethylated lambda DNA (Promega) with M.SssI enzyme (NEB) treatment. 2-kb-unmodified spike-in was produced by PCR amplification from the pNIC28-Bsa4 plasmid (Addgene, cat no. 26103). Synthetic spike-in with 5mC and 5hmC modifications was produced by annealing and extension of one oligo containing 5mC and another oligo containing 5hmC (IDT). 5fC spike-in was produced by an annealing and extension method with 5-formylcytidine-5ʹ-triphosphate (5-fCTP, TriLink BioTechnologies). 5caC spike-in was produced by PCR amplification from the pNIC28-Bsa4 plasmid, then methylated with M.SssI enzyme (NEB) and oxidized with two rounds of mTet1CD treatment. Detailed preparation protocols and sequences of the spike-in DNA can be found in previous publication^15^ and Supplementary Table 3.

### mESCs culture and gDNA extraction

E14 mESCs were gifted from Professor Skirmantas Kriaucionis and cultured on gelatin-coated plates in Dulbecco’s Modified Eagle Medium (DMEM) (Invitrogen) supplemented with 15% FBS (GIBCO), 2 mM l-glutamine (Gibco), 1% non-essential amino acids (Gibco), 1% penicillin/streptavidin (Gibco), 0.1 mM β-mercaptoethanol (Sigma), 1000 units/mL LIF (Millipore), 1 µM PD0325901 (Stemgent), and 3 µM CHIR99021 (Stemgent). mESCs were maintained at 37 °C and 5% CO_2_ and passaged every 2 days. The gDNA was prepared by cell harvesting with centrifugation for 5 min at 1000 × *g* and room temperature, and DNA extraction with Quick-DNA Plus kit (Zymo Research) according to the manufacturer’s protocol.

### Expression and purification of mTet1CD

mTet1CD was expressed in Expi293F cells from mTet1CD insert (NM_001253857.2, 4371-6392) with N-terminal Flag-tag cloned into pcDNA3-Flag between KpnI and BamH1 restriction sites^15^. The cells were grown for 48 h at 37 °C, 170 r.p.m., and 5% CO_2_, then harvested by centrifugation, re-suspended in the lysis buffer containing 50 mM Tris–Cl pH 7.5, 500 mM NaCl, 1× cOmplete Protease Inhibitor Cocktail, 1 mM PMSF, 1% Triton X-100, and incubated on ice for 20 min. The cell lysate was then clarified by centrifugation for 30 min at 30,000 × *g* and 4 °C. ANTI-FLAG M2 Affinity Gel was used to purify the supernatant and eluted with buffer containing 20 mM HEPES pH 8.0, 150 mM NaCl, 0.1 mg/mL 3× Flag peptide, 1× cOmplete Protease Inhibitor Cocktail, 1 mM PMSF. The collected fractions were concentrated and buffer exchanged to the final buffer containing 20 mM HEPES pH 8.0, 150 mM NaCl, and 1 mM dithiothreitol, then mixed with glycerol (30% v/v) for storage at −80 °C.

### Preparation of mESCs gDNA and sequencing library construction

mESCs gDNA was spiked with 0.5% of methylated lambda DNA, 0.025% of 2-kb-unmodified and 0.025% of 2-kb-caC spike-in controls. For CAPS approach, gDNA was fragmented by Covaris M220 instrument and size-selected to 200–400 bp using Ampure XP beads (Beckman Coulter). For other approaches, gDNA was fragmented and size-selected to 300–500 bp; 0.01% of synthetic oligo with N5mCNN/N5hmCNN sequences and 0.01% of synthetic oligo with 5fC modifications were added after size-selection. One-hundred nanograms of fragmented DNA was used for end-repair/A-tailing and ligation of NEBNext Adaptor (NEB) with KAPA Hyper kit (KAPA) according to the manufacturer’s protocol. The uracil in the loop of NEBNext Adaptor was removed by adding 3 μL of USER enzyme (NEB) to the ligation reaction and incubating for 15 min at 37 °C. Then the reaction was purified with 0.8× Ampure XP beads according to the manufacturer’s protocol. For CAPS approach, 80% acetonitrile:H_2_O was used instead of 80% ethanol:H_2_O during the beads purification step.

### TAPS with βGT blocking (TAPSβ)

Ligated DNA was added to a 50-μL reaction containing 50 mM HEPES buffer (pH 8), 25 mM MgCl_2_, 200 μM UDP-Glc (NEB), and 10 U of βGT (Thermo Fisher) for 1 h at 37 °C. 5hmC-blocked DNA was purified with Ampure XP and then incubated in 50 µL oxidation reaction containing 50 mM HEPES buffer (pH 8.0), 100 µM ammonium iron (II) sulfate, 1 mM α-ketoglutarate, 2 mM ascorbic acid, 1 mM dithiothreitol, 100 mM NaCl, 1.2 mM ATP, and 4 μM mTet1CD for 80 min at 37 °C. Then 0.8 U of Proteinase K (NEB) was added to the reaction and incubated for 1 h at 50 °C. Oxidized DNA was purified with Ampure XP beads and then input into another round of TET oxidation in order to achieve complete oxidation. The double-oxidized DNA was added to a 50-μL reaction containing 600 mM NaAc (pH = 4.3) and 1 M pyridine borane (Alfa Aesar). The reaction was incubated at 37 °C and 850 r.p.m. in a ThermoMixer (Eppendorf) for 16 h and purified by Zymo-IC column (Zymo Research) with Oligo Binding Buffer (Zymo Research).

### Chemical-assisted pyridine borane sequencing (CAPS)

Potassium ruthenate (K_2_RuO_4_) was prepared as previously described by Zeng et al.^23^ and stored at −20 °C in a refrigerator as 10× oxidant; 2 M pic-borane (Sigma) was prepared by dissolving the solid in EtOH. Before 5hmC oxidation, ligated DNA was purified with Micro Bio-Spin P-6 SSC column (Bio-Rad, washed five times with water before use). The purified DNA was denatured in 20 μL solution containing 0.05 M NaOH for 30 min at 37 °C; 10× oxidant was diluted to 1× with distilled water and 2.5 μL of 1× oxidant was added to the denatured DNA. The oxidation reaction was incubated at 37 °C and 850 r.p.m. in a ThermoMixer for 1 h. Then additional 2.5 μL of 1× oxidant was added to the same reaction and incubated at 37 °C and 850 r.p.m. in a ThermoMixer for another hour. The oxidized DNA was purified by a Bio-Rad Micro Bio-Spin P-6 SSC column, and added to a reaction containing 0.6 M MES (Sigma, pH 5.2) and 0.2 M pic-borane. The reaction was incubated at 37 °C and 850 r.p.m. in a ThermoMixer for 2 h and purified by Zymo-IC column with Oligo Binding Buffer.

### Quantification of 5mC, 5hmC, and 5fC level by HPLC-MS/MS

Control and oxidized gDNA samples were digested into nucleosides by 2 U of Nuclease P1 (Sigma-Aldrich) and 10 nM deaminase inhibitor erythro-9-amino-β-hexyl-α-methyl-9H-purine-9-ethanol hydrochloride (Sigma-Aldrich) at 37 °C for overnight and then 6 U of alkaline phosphatase (Sigma-Aldrich) and 0.5 U of phosphodiesterase I (Sigma-Aldrich) at 37 °C for 3 h. After filtering with Amicon Ultra-0.5 mL 10K centrifugal filters (Merck Millipore), the digested samples were subjected to a ZORBAX Eclipse Plus C18 column (2.1 × 150 mm^2^, 1.8-μm, Agilent). HPLC–MS/MS analysis was carried out with 1290 Infinity LC Systems (Agilent) coupled with a 6495B Triple Quadrupole Mass Spectrometer (Agilent). Detailed HPLC-MS/MS program could be found in previous publication^15^.

### Pyridine borane sequencing (PS)

Ligated DNA was added to a 50-μL reaction containing 0.6 M NaAc (pH = 4.3) and 1 M pyridine borane. The reaction was incubated at 37 °C and 850 r.p.m. in a ThermoMixer for 16 h and purified by Zymo-IC column with Oligo Binding Buffer.

### Pyridine borane sequencing for carboxylcytosine (PS-c)

Ligated DNA was added to a 50-μL reaction containing 10 mM *O*-ethylhydroxylamine (Aldrich) and 100 mM MES buffer (pH 5.0). The reaction was incubated at 37 °C and 850 r.p.m. for 4 h in a ThermoMixer and purified with Ampure XP beads. 5fC-blocked DNA was then added to a 50-μL reaction containing 0.6 M NaAc (pH = 4.3) and 1 M pyridine borane. The reaction was incubated at 37 °C and 850 r.p.m. in a ThermoMixer for 16 h and purified by Zymo-IC column with Oligo Binding Buffer.

### PCR amplification of converted DNA and sequencing

Converted DNA was amplified with KAPA HiFi HotStart Uracil+ ReadyMix PCR Kit (KAPA) for 4 cycles according to the manufacturer’s protocol with minor modification. Dual index primers in NEBNext Multiplex Oligos for Illumina were used instead of the Library Amplification Primer Mix. The PCR product was purified with 1× Ampure XP beads and quantified with Qubit dsDNA HS Assay Kit (ThermoFisher). When starting with 100 ng of fragmented DNA for library construction, typical final library yield should be >30 nM after 4 cycles of PCR amplification. Libraries were sequenced on NovaSeq 6000 (150 bp paired end) with no PhiX added.

### Data preprocessing

Sequencing reads were trimmed with Trim Galore! v0.3.1 (https://www.bioinformatics.babraham.ac.uk/projects/trim_galore/) to remove adaptors and low-quality bases. Trimmed reads were mapped to a genome combining spike-in sequences and the mm9 mouse genome using BWA mem v.0.7.12 (ref. ^35^). PCR duplicates were removed using MarkDuplicate function of Picard v2.3.0 (http://broadinstitute.github.io/picard/). Reads with MAPQ < 10 were excluded from methylated site calling. Modified bases were called by asTair v3.3.1 (ref. ^15^). Raw signals were calculated as the ratio between C and C+T at each site. Regions known to be prone to mapping artifacts (https://sites.google.com/site/anshulkundaje/projects/blacklists)^36,37^ and known single nucleotide variants (http://epigenetics.hugef-research.org/data.php)^38^ of the E14 cell line were used to exclude those overlapping sites from subsequent analysis. The mapping rate was calculated as the ratio between the number of properly mapped read pairs (MAPQ > 10) and the number of trimmed read pairs by Samtools^39^. The base quality was visualized by the phred function of asTair^15^.

### Published datasets

We used the following published datasets: TAPS data and WGBS data (GSE112520)^15^, RRoxBS-seq data (GSM1364069)^21^, oxBS-seq data (GSE112875)^22^, TAB-seq data (GSE36173)^10^, and ACE-seq data (GSE116016)^13^. The TAB-seq data were reprocessed to obtain the full list of modified and unmodified sites. The sequencing reads were downloaded and trimmed by Trim Galore! v0.3.1 (https://www.bioinformatics.babraham.ac.uk/projects/trim_galore/). The trimmed reads were aligned to mm9 using Bismark v0.18.1 (ref. ^40^) and Bowtie v2.2.1 (ref. ^41^). PCR duplicates were removed from the mapped bam file using MarkDuplicate function of Picard v2.3.0 (http://broadinstitute.github.io/picard/). The reads with over three non-conversion sites were filtered using the filter_non_conversion function of bismark as previously described^10^. The methylation sites were called by bismark_methylation_extractor and masked by intersectBed (Bedtools v2.25.0)^42^ to remove sites in regions known to be prone to mapping artifacts.

### Pairwise comparisons of TAPSβ

Replicate one of RRoxBS-seq data was used due to the highest number of reads among the four replicates^21^. The three replicates of whole-genome oxBS-seq^22^ results were pooled together for the correlation analysis. Sites with a minimal coverage of ten reads were used for the correlation analysis between TAPSβ and oxBS-seq. The Pearson correlation coefficient (Pearson’s *r*) was calculated by using R function cor. The scatterplots with smoothed densities color representation were visualized using function smoothScatter in R.

### Coverage analysis of CAPS and ACE-seq

The CpG island annotation was downloaded from UCSC^43^. Each CpG island was evenly binned into ten windows. The 4-kb flanking regions were binned into 20 windows. The coverage was defined as the sum of modified and unmodified reads at each site. The average coverage was calculated by Bedtools map^42^. Given that the overall coverage of CAPS was higher than ACE-seq, the coverage at each site was normalized by the ratio of overall coverage between the two datasets.

### Pairwise comparisons of CAPS

To compare CAPS with ACE-seq and TAB-seq, the raw 5hmCG signals, i.e. C/(C+T), were calculated within 10-kb genomic bins (Fig. 2f) as previously defined^13^. The 10-kb raw signal of TAPS−TAPSβ subtraction was calculated as the average estimated 5hmC levels from the maximum likelihood methylation levels (MLML) output.

### Estimation of 5hmC using maximum likelihood

To estimate 5hmC levels from TAPS and TAPSβ, the MLML estimation method^19^ was applied on sites with a minimum coverage of 5. The sites with at least one conflict were excluded from subsequent analysis. The average levels of 5mC and 5hmC estimated by MLML were tiled by 1-kb bins (Fig. 3a).

### Genomic view

To view the methylation levels on genomes, the methylation calling output was transferred to the bigwig format by bedGraphToBigWig^44^ and visualized by the Integrative Genomics Viewer^45^ on the mm9 genome.

### Statistical test of 5hmC

We used the binomial test^10^ to call 5hmC at sites with the minimal coverage of five reads. The probability *p* of the binomial distribution was the false-positive rate (0.0072) of CAPS, calculated from the unmodified control DNA (Fig. 2c). Cytosines with Benjamini–Hochberg (BH) adjusted *p*-value <0.05 were used for downstream analysis.

### Quantifying enrichment of 5hmCGs in regulatory elements

The list of putative genomic regulatory elements was downloaded (https://github.com/gireeshkbogu/chromatin_states_chromHMM_mm9)^27^. This list was predicted based on the ENCODE data^26^ by ChromHMM^25^. The high-confidence 5hmCG sites (BH-adjusted *p*-value < 0.05 and coverage ≥5 reads) were annotated using bedtools intersect. The number of 5hmCG sites falling into each category was counted (Fig. 3c). To investigate the enrichment of 5hmCG in each element class, a set of CG sites was sampled for ten times to generate a background distribution of CG sites across element categories. The number of 5hmCGs or random CGs was normalized by the genomic coverage of corresponding regulatory elements.

### Genome-wide analysis of PS and PS-c

The histone modification ChIP-seq data were downloaded from the ENCODE project^34^: H3K4me1 (GSM1000121) and H3K4me3 (GSM1000124). The prediction result of genomic regulatory elements^27^ was downloaded as described above. The centers of broad peaks or predicted regions were used. Average modification levels were calculated by tiling the left and right flanking 3 kb regions into 100-bp bins. The profiles were visualized by deepTools 3.3.0 (ref. ^46^).

### Reporting summary

Further information on research design is available in the Nature Research Reporting Summary linked to this article.

## Supplementary information

Supplementary Information

Reporting Summary

## Data Availability

All sequencing data of this study are deposited at the Gene Expression Omnibus (accession: GSE155613). Published data used in this study include TAPS data and WGBS data (GSE112520)^15^, RRoxBS-seq data (GSM1364069)^21^, oxBS-seq data (GSE112875)^22^, TAB-seq data (GSE36173)^10^ and ACE-seq data (GSE116016)^13^, H3K4me1 ChIP-seq data (GSM1000121), and H3K4me3 ChIP-seq data (GSM1000124)^34^. All relevant additional data have been published with the manuscript, either as part of the main text or in the supplement.

## Code availability

The analysis scripts are available at https://github.com/zhiyhu/CAPS-paper; 10.5281/zenodo.4321048 (ref. ^47^).
