# Supplementary material for: Subtraction-free and bisulfite-free specific sequencing of 5-methylcytosine and its oxidized derivatives at base resolution
Source: Nat Commun. 2021 Jan 27;12:618. doi: 10.1038/s41467-021-20920-2 (PMC7840749; doi:10.1038/s41467-021-20920-2)
Supplement: Supplementary file 1 — Supplementary Information [file 41467_2021_20920_MOESM1_ESM.pdf]

Supporting Information for

Subtraction-free and bisulfite-free specific sequencing of 5-methylcytosine and its oxidized derivatives at base resolution

Yibin Liu[1,2,5,*], Zhiyuan Hu[3,4,*], Jingfei Cheng[1,2], Paulina Siejka-Zielińska[1,2], Jinfeng Chen[1,2], Masato Inoue[1,2], Ahmed Ashour Ahmed[3,4], Chun-Xiao Song[1,2,†]

[†]Corresponding author. Email: chunxiao.song@ludwig.ox.ac.uk

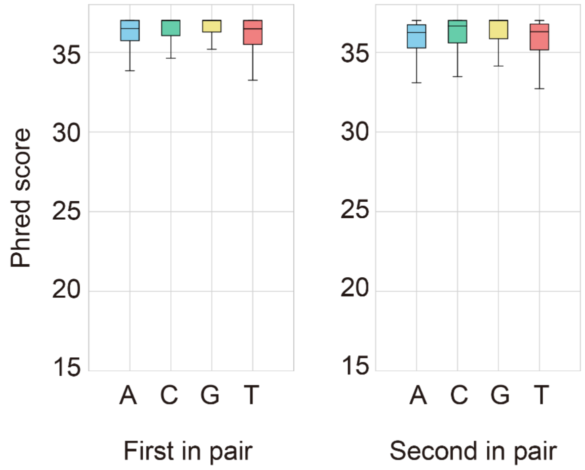

Supplementary Figure 1. TAPSβ sequencing quality scores per base for the first and second reads in all sequenced read pairs. Boxplots show the medians, upper and lower fourth quantiles and non-outlier extreme values (n = 10 million random sequencing reads).

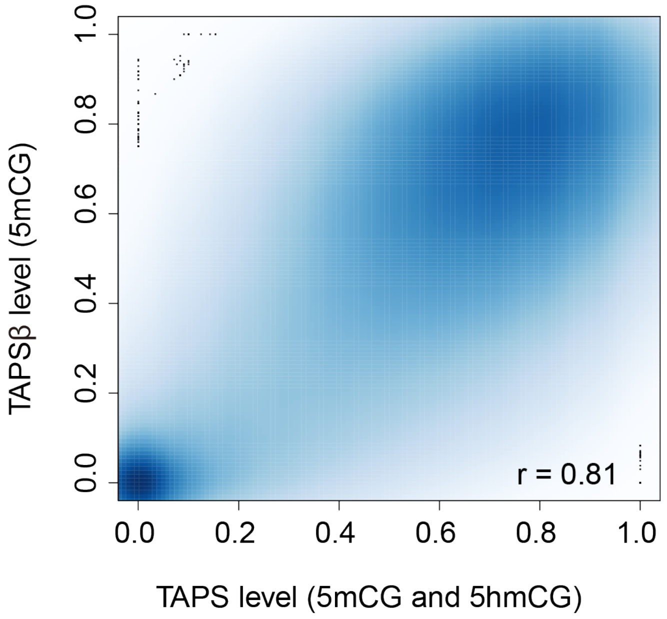

Supplementary Figure 2. Correlation analysis between TAPSβ and TAPS at CpGs with the minimal depth of 5. The Pearson's r is shown at the right bottom.

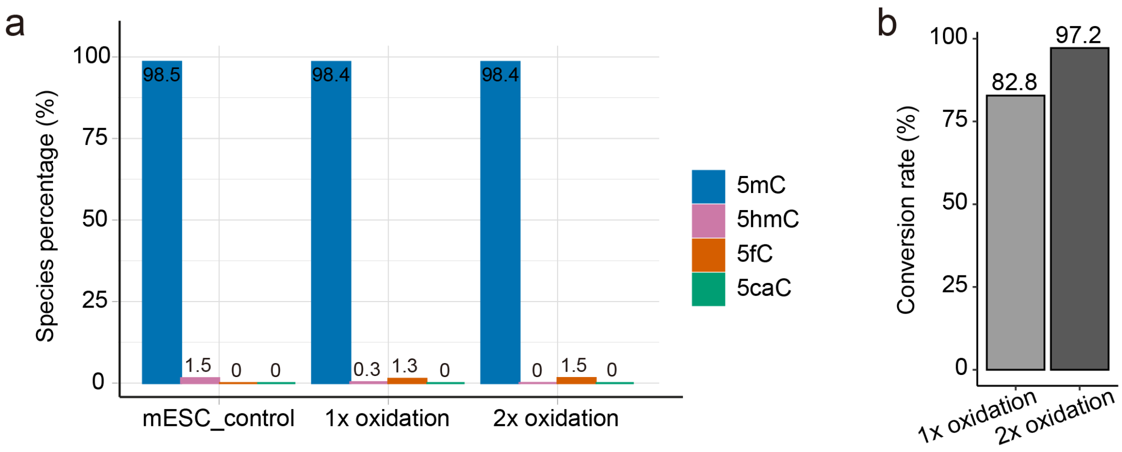

Supplementary Figure 3. Two rounds of K_2RuO_4 oxidation achieved more complete 5hmC to 5fC conversion than one round. (a) HPLC–MS/MS quantification of relative modification levels in the mESCs genomic DNA control[1], after one round K_2RuO_4 (1x) oxidation and after two rounds K_2RuO_4 (2x) oxidation. 5fC or 5caC was not detected in the control sample. Experiment was performed once. (b) Conversion rate of 5hmC to 5fC was calculated by relative MS signal intensity. Conversion rate = $Intensity_{5fC}$ / ($Intensity_{5fC}$ + $Intensity_{5hmC}$).

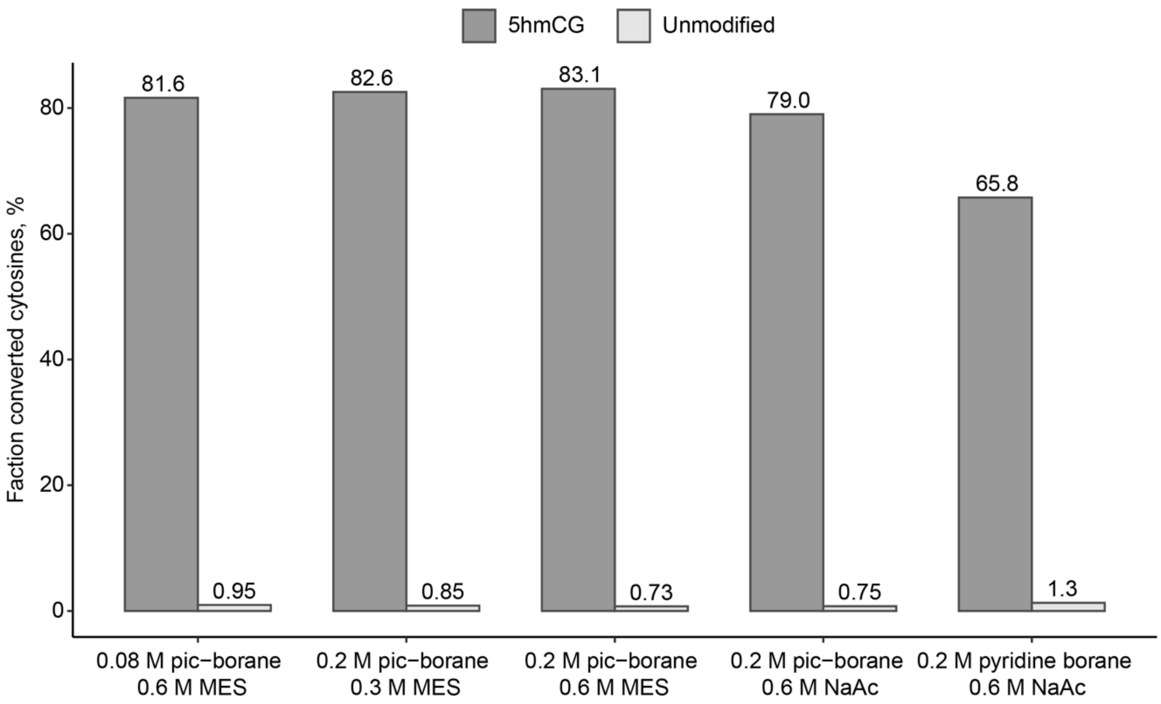

Supplementary Figure 4. The conversion rates at 5hmCG position from synthetic spike-in with 5mC and 5hmC modifications and false positive rates from 2kb-unmodified spike-in under different CAPS conditions. Ligated DNA was treated with 2× K_2RuO_4 oxidation and then incubated with pic-borane or pyridine borane and MES (pH 5.2) or NaAc (pH 4.3) buffer at 37°C for 2 h.

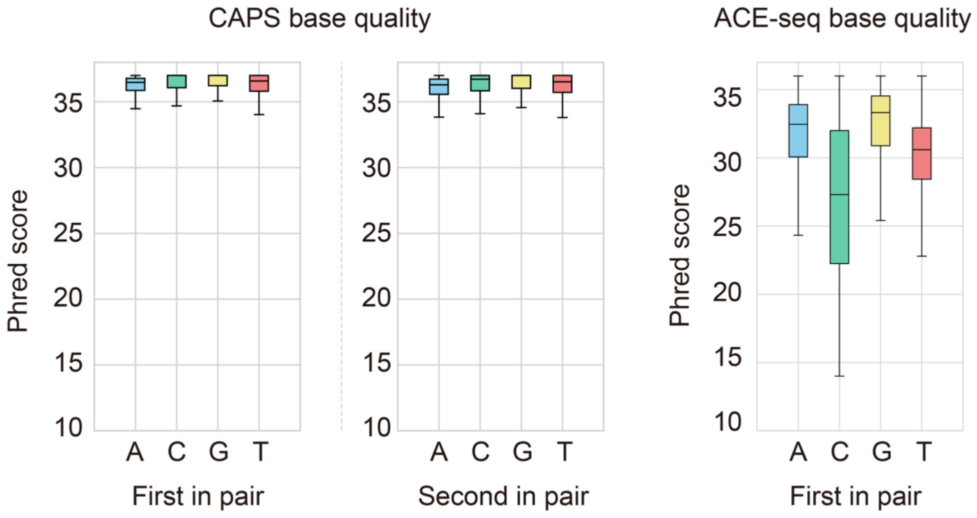

Supplementary Figure 5. Comparison of sequencing base quality between CAPS and ACE-seq. CAPS (sequenced in pair-end mode) showed good sequencing quality scores per base for the first and second reads in all sequenced read pairs while ACE-seq (sequenced in single-end mode) showed lower sequencing quality scores per base for the first read. Boxplots show the medians, upper and lower fourth quantiles and non-outlier extreme values (n = 10 million random sequencing reads).

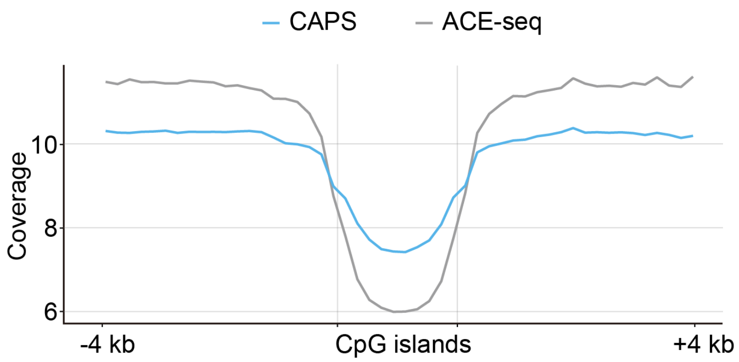

Supplementary Figure 6. Average sequencing coverage depth of CAPS and ACE-seq at all CpG islands (CGI) and 4-kb flanking regions.

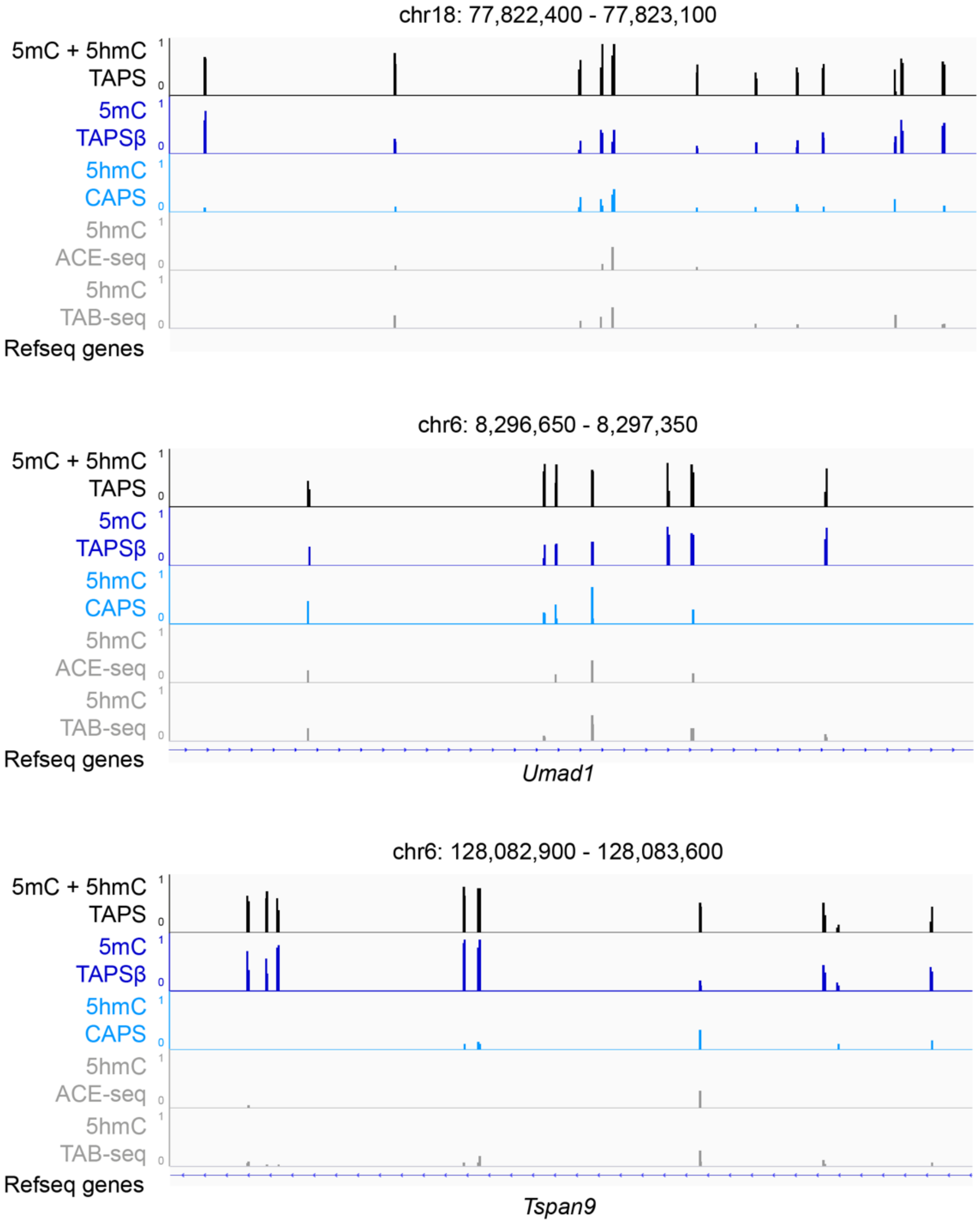

Supplementary Figure 7. IGV screenshots show the consistency between CAPS and other techniques at multiple genomic loci. The y-axis represents the modification level, and the x-axis indicates the genomic positions. The range of genomic regions is shown as subtitles.

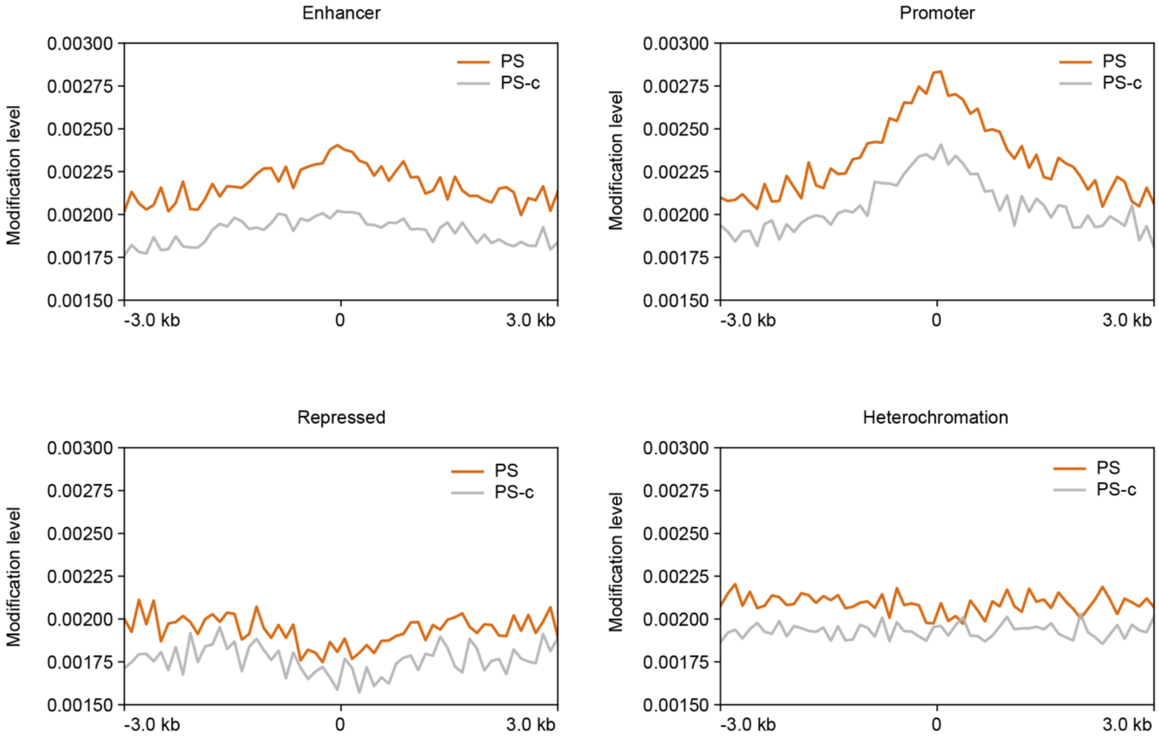

Supplementary Figure 8. 5fC/5caC signals in putative genomic regulatory regions (enhancers, promoters, repressed and heterochromatin) and their flanking regions in mESC E14. In the x-axis, 0 represents the center of the peak.

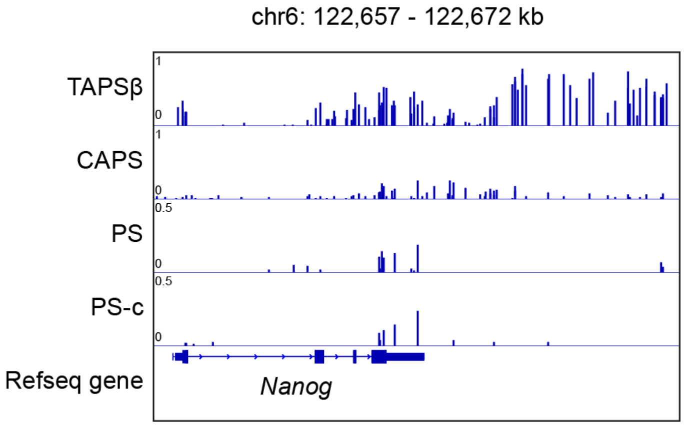

Supplementary Figure 9. IGV screenshot shows the 5fC/5caC modification on *Nanog*. The y-axis represents the modification level, and the x-axis indicates the genomic positions. Only CpGs with the minimum depth of 10 are shown.

Supplementary Table 1. Conversion rates of unmodified C, 5mC, 5hmC, 5fC, and 5caC in TAPS, TAPSβ, CAPS, PS and PS-c.

Conversion	C	5mC-λ	5mC	5hmC	5fC	5caC
TAPS[1]	0.23%	96.5%	97.3%	89.1%	NA	NA
TAPSβ	0.24%	97.6%	94.8%	1.9%	84.9%	94.4%
CAPS	0.72%	0.38%	0.62%	83.1%	85.2%	96.2%
PS	0.27%	0.10%	0.32%	1.8%	76.8%	93.8%
PS-c	0.22%	0.10%	0.22%	1.4%	15.2%	95.3%

Conversion rates were calculated based on the corresponding spike-in controls. C: 2kb-unmodified spike-in; 5mC-λ: CpG-methylated lambda DNA; 5mC and 5hmC: synthetic spike-in with 5mC and 5hmC modifications; 5fC: 5fC spike-in; 5caC: 5caC spike-in. Conversion rates of 5fC and 5caC are not available (NA) for published TAPS data.

Supplementary Table 2. Alignment and deduplication metrics of sequencing data.

	Assays	Total sequenced reads or read pairs	Mapping rate (%)	Duplication rate (%)	Usable rate (%)
5mC	TAPSβ	405,560,101	90.7	20.8	71.9
	RRoxBS-seq[2]	62,876,031	66.2-68.2	NA	NA
	oxBS-seq[3]	192,166,218	21.4-26.1	12.1-18.1	18.6-21.4
5hmC	CAPS	336,706,866	90.7	24.2	68.8
	TAB-seq[4]	998,337,691	53.4	55.5	23.8
	ACE-seq[5]	476,595,789	71.8	16.2	60.2
5fC	PS	272,591,664	90.4	20.0	72.3
5caC	PS-c	295,571,425	90.4	20.7	71.8

Supplementary Table 3. Primer sequences for 5fC and 5caC spike-ins.

Spike-in	Sequence (5’-3’)
5fC (modified C marked in yellow)	Produced by primer extension with Klenow exo- and dfCTP on two annealed DNA oligos:<br>AGAGAGCAAGCCGGCTATAGATGCTACGTACAGTAGCAGCTGATCAAGACTGCTAAGGC<br>CACAACCAGTTGGCG and<br>ACTCTCACTCTCACCTCCATCTTACTTGTCTACCGAATCCTCACGTACTCACGATTCGACC<br>ATCCACATTCGCCAACUGGUTGUGGCCTT<br><br>Final spike-in sequence:<br>AGAGAGCAAGCCGGCTATAGATGCTACGTACAGTAGCAGCTGATCAAGACTGCTAAGGC<br>CACAACCAGTTGGCGAATGTGGATGGTCGAATCGTGAGTACGTGAGGATTCGGTAGACA<br>AGTAAGATGGAGGTGAGAGTGAGAGT
5caC (modified C marked in blue)	Phusion HF PCR amplification with:<br>Forward primer: ACTGGAACAACACTCAACCCTA<br>Reverse primer: AGAGCAGATTGTACTGAGAGTG<br><br>Final spike-in sequence:<br>ACTGGAACAACACTCAACCCTATCTCGGTCTATTCTTTTGATTTATAAGGGATTTTGCCGA<br>TTTCGGCCTATTGGTTAAAAAATGAGCTGATTTAACAAAAATTTAACGCGAATTTTAACAA<br>AATATTAACGTTTACAATTTCAGGTGGCACTTTTCGGGGAAATGTGCGCGGAACCCCTAT<br>TTGTTTATTTTTCTAAATACATTCAAATATGTATCCGCTCATGAATTAATTCTTAGAAAAACT<br>CATCGAGCATCAAATGAAACTGCAATTTATTCATATCAGGATTATCAATACCATATTTTTGA<br>AAAAGCCGTTTCTGTAATGAAGGAGAAAACTCACCGAGGCAGTTCCATAGGATGGCAAG<br>ATCCTGGTATCGGTCTGCGATTCCGACTCGTCCAACATCAATACAACCTATTAATTTCCCC<br>TCGTCAAAAATAAGGTTATCAAGTGAGAAATCACCATGAGTGACGACTGAATCCGGTGAG<br>AATGGCAAAAGTTTATGCATTTCTTTCCAGACTTGTTCAACAGGCCAGCCATTACGCTCG<br>TCATCAAAATCACTCGCATCAACCAAACCGTTATTCATTCGTGATTGCGCCTGAGCGAGA<br>CGAAATACGCGATCGCTGTTAAAAGGACAATTACAAACAGGAATCGAATGCAACCGGCG<br>CAGGAACACTGCCAGCGCATCAACAATATTTTCACCTGAATCAGGATATTCTTCTAATACC<br>TGGAATGCTGTTTTCCCGGGGATCGCAGTGGTGAGTAACCATGCATCATCAGGAGTACG<br>GATAAAATGCTTGATGGTCGGAAGAGGCATAAATTCCGTCAGCCAGTTTAGTCTGACCAT<br>CTCATCTGTAACATCATTGGCAACGCTACCTTTGCCATGTTTCAGAAACAACTCTGGCGC<br>ATCGGGCTTCCCATACAATCGATAGATTGTCGCACCTGATTGCCCGACATTATCGCGAGC<br>CCATTTATACCCATATAAATCAGCATCCATGTTGGAATTTAATCGCGGCCTAGAGCAAGA<br>CGTTTCCCGTTGAATATGGCTCATAACACCCCTTGTATTACTGTTTATGTAAGCAGACAGT<br>TTTATTGTTCATGACCAAAATCCCTTAACGTGAGTTTTCGTTCCACTGAGCGTCAGACCCC<br>GTAGAAAAGATCAAAGGATCTTCTTGAGATCCTTTTTTTCTGCGCGTAATCTGCTGCTTGC<br>AAACAAAAAAACCACCGCTACCAGCGGTGGTTTGTTTGCCGGATCAAGAGCTACCAACT<br>CTTTTTCCGAAGGTAACTGGCTTCAGCAGAGCGCAGATACCAAATACTGTCCTTCTAGTG<br>TAGCCGTAGTTAGGCCACCACTTCAAGAACTCTGTAGCACCGCCTACATACCTCGCTCTG<br>CTAATCCTGTTACCAGTGGCTGCTGCCAGTGGCGATAAGTCGTGTCTTACCGGGTTGGA<br>CTCAAGACGATAGTTACCGGATAAGGCGCAGCGGTCGGGCTGAACGGGGGGTTCGTGC<br>ACACAGCCCAGCTTGGAGCGAACGACCTACACCGAACTGAGATACCTACAGCGTGAGCT<br>ATGAGAAAGCGCCACGCTTCCCGAAGGGAGAAAGGCGGACAGGTATCCGGTAAGCGGC<br>AGGGTCGGAACAGGAGAGCGCACGAGGGAGCTTCCAGGGGGAAACGCCTGGTATCTTT<br>ATAGTCCTGTCGGGTTTCGCCACCTCTGACTTGAGCGTCGATTTTTGTGATGCTCGTCAG<br>GGGGGCGGAGCCTATGGAAAAACGCCAGCAACGCGGCCTTTTTACGGTTCCTGGCCTTT<br>TGCTGGCCTTTTGCTCACATGTTCTTTCCTGCGTTATCCCCTGATTCTGTGGATAACCGTA<br>TTACCGCCTTTGAGTGAGCTGATACCGCTCGCCGCAGCCGAACGACCGAGCGCAGCGA<br>GTCAGTGAGCGAGGAAGCGGAAGAGCGCCTGATGCGGTATTTTCTCCTTACGCATCTGT<br>GCGGTATTTCACACCGCATATATGGTGCACTCTCAGTACAATCTGCTCT

References

1. Liu, Y. et al. Bisulfite-free direct detection of 5-methylcytosine and 5-hydroxymethylcytosine at base resolution. *Nat. Biotechnol.* **37**, 424-429 (2019).
2. Booth, M.J., Marsico, G., Bachman, M., Beraldi, D. & Balasubramanian, S. Quantitative sequencing of 5-formylcytosine in DNA at single-base resolution. *Nat Chem* **6**, 435-440 (2014).
3. Liu, C. et al. DNA 5-Methylcytosine-Specific Amplification and Sequencing. *J. Am. Chem. Soc.* **142**, 4539-4543 (2020).
4. Yu, M. et al. Base-resolution analysis of 5-hydroxymethylcytosine in the mammalian genome. *Cell* **149**, 1368-1380 (2012).
5. Schutsky, E.K. et al. Nondestructive, base-resolution sequencing of 5-hydroxymethylcytosine using a DNA deaminase. *Nat. Biotechnol.* **36**, 1083-1090 (2018).
