# Supplementary material for: Subtraction-free and bisulfite-free specific sequencing of 5-methylcytosine and its oxidized derivatives at base resolution
Source: Nat Commun. 2021 Jan 27;12:618. doi: 10.1038/s41467-021-20920-2 (PMC7840749; doi:10.1038/s41467-021-20920-2)
Supplement: Supplementary file 2 — Reporting Summary [file 41467_2021_20920_MOESM2_ESM.pdf]

nature research

Corresponding author(s): Chun-Xiao Song

Last updated by author(s): Dec 20, 2020

Reporting Summary

Nature Research wishes to improve the reproducibility of the work that we publish. This form provides structure for consistency and transparency in reporting. For further information on Nature Research policies, see Authors & Referees and the Editorial Policy Checklist .

Statistics

For all statistical analyses, confirm that the following items are present in the figure legend, table legend, main text, or Methods section.

n/a	Confirmed	
☐	☒	The exact sample size (*n*) for each experimental group/condition, given as a discrete number and unit of measurement
☐	☒	A statement on whether measurements were taken from distinct samples or whether the same sample was measured repeatedly
☐	☒	The statistical test(s) used AND whether they are one- or two-sided<br>*Only common tests should be described solely by name; describe more complex techniques in the Methods section.*
☒	☐	A description of all covariates tested
☐	☒	A description of any assumptions or corrections, such as tests of normality and adjustment for multiple comparisons
☐	☒	A full description of the statistical parameters including central tendency (e.g. means) or other basic estimates (e.g. regression coefficient) AND variation (e.g. standard deviation) or associated estimates of uncertainty (e.g. confidence intervals)
☐	☒	For null hypothesis testing, the test statistic (e.g. *F*, *t*, *r*) with confidence intervals, effect sizes, degrees of freedom and *P* value noted<br>*Give P values as exact values whenever suitable.*
☒	☐	For Bayesian analysis, information on the choice of priors and Markov chain Monte Carlo settings
☒	☐	For hierarchical and complex designs, identification of the appropriate level for tests and full reporting of outcomes
☐	☒	Estimates of effect sizes (e.g. Cohen's *d*, Pearson's *r*), indicating how they were calculated

Our web collection on statistics for biologists contains articles on many of the points above.

Software and code

Policy information about availability of computer code

Data collection	No software was used.
Data analysis	The analysis scripts are available at https://github.com/zhiyhu/CAPS-paper. We also used: Trim Galore v0.3.1, BWA v.0.7.12, Picard v2.3.0, asTair v3.3.1, Bismark v0.18.1, Bowtie v2.2.1, Bedtools v2.25.0, Integrative Genomics Viewer and deepTools 3.3.0. Further details are described in the Methods section.

For manuscripts utilizing custom algorithms or software that are central to the research but not yet described in published literature, software must be made available to editors/reviewers. We strongly encourage code deposition in a community repository (e.g. GitHub). See the Nature Research guidelines for submitting code & software for further information.

Data

Policy information about availability of data

All manuscripts must include a data availability statement. This statement should provide the following information, where applicable:

- Accession codes, unique identifiers, or web links for publicly available datasets
- A list of figures that have associated raw data
- A description of any restrictions on data availability

All sequencing data of this study are deposited at the Gene Expression Omnibus (accession: GSE155613 [https://www.ncbi.nlm.nih.gov/geo/query/acc.cgi?acc=GSE155613]). Published data used in this study include TAPS data and WGBS data (GSE112520 [https://www.ncbi.nlm.nih.gov/geo/query/acc.cgi?acc=GSE112520])15, RRoxBS-seq data (GSM1364069 [https://www.ncbi.nlm.nih.gov/geo/query/acc.cgi?acc=GSM1364069])21, oxBS-seq data (GSE112875 [https://www.ncbi.nlm.nih.gov/geo/query/acc.cgi?acc=GSE112875])22, TAB-seq data (GSE36173 [https://www.ncbi.nlm.nih.gov/geo/query/acc.cgi?acc=GSE36173])10 and ACE-seq data (GSE116016 [https://www.ncbi.nlm.nih.gov/geo/query/acc.cgi?acc=GSE116016])13, H3K4me1 ChIP-seq data (GSM1000121 [https://www.ncbi.nlm.nih.gov/geo/query/acc.cgi?acc=GSM1000121]) and H3K4me3 ChIP-seq data (GSM1000124 [https://www.ncbi.nlm.nih.gov/geo/query/acc.cgi?acc=GSM1000124]). All relevant additional data have been published with the manuscript, either as part of the main text or in the supplement.

Field-specific reporting

Please select the one below that is the best fit for your research. If you are not sure, read the appropriate sections before making your selection.

- [x] Life sciences
- [ ] Behavioural & social sciences
- [ ] Ecological, evolutionary & environmental sciences

For a reference copy of the document with all sections, see nature.com/documents/nr-reporting-summary-flat.pdf

Life sciences study design

All studies must disclose on these points even when the disclosure is negative.

Sample size	No sample-size calculation was performed as we are using the same mESCs E14 genomic DNA to demonstrate the methods.
Data exclusions	No data were excluded from the analyses.
Replication	No replication of experiment was performed on the sequencing because one whole-genome sequencing run would generate multiple data points in each condition for the analysis.
Randomization	No randomization was used for the sampling as we are using the same mESCs E14 genomic DNA to demonstrate the methods.
Blinding	Investigators were not blinded to sample allocation during data collection. The aim for this study is methodology development and the blinding is not necessary.

Reporting for specific materials, systems and methods

We require information from authors about some types of materials, experimental systems and methods used in many studies. Here, indicate whether each material, system or method listed is relevant to your study. If you are not sure if a list item applies to your research, read the appropriate section before selecting a response.

Materials & experimental systems

n/a	Involved in the study	
[x]	[ ]	Antibodies
[ ]	[x]	Eukaryotic cell lines
[x]	[ ]	Palaeontology
[x]	[ ]	Animals and other organisms
[x]	[ ]	Human research participants
[x]	[ ]	Clinical data

Methods

n/a	Involved in the study	
[x]	[ ]	ChIP-seq
[x]	[ ]	Flow cytometry
[x]	[ ]	MRI-based neuroimaging

Eukaryotic cell lines

Policy information about cell lines

Cell line source(s)	Mouse embryonic stem cells E14 were gifted from Professor Skirmantas Kriaucionis (original source from Prof. Adrian Bird).
Authentication	Mouse embryonic stem cells E14 were not authenticated.
Mycoplasma contamination	Mouse embryonic stem cells E14 were negative for mycoplasma test.
Commonly misidentified lines (See ICLAC register)	No commonly misidentified cell lines were used.
